# Primary Central Nervous System Lymphoma Presenting as Multifocal Brain Abscesses

**DOI:** 10.7759/cureus.12068

**Published:** 2020-12-14

**Authors:** Sai Liang, Jia Xu Lim, Hwei Yee Lee, Sharon YY Low

**Affiliations:** 1 Neurosurgery, National Neuroscience Institute, Singapore, SGP; 2 Pathology, Tan Tock Seng Hospital, Singapore, SGP

**Keywords:** brain abscess, central nervous system lymphoma, immunosuppressant

## Abstract

A previously well female presented with a history of progressive functional decline. She had a known history of generalized seropositive myasthenia gravis on long-term mycophenolate mofetil and pyridostigmine. MRI of her brain reported multiple cerebral abscesses based on radiological features. The patient was commenced on intravenous antibiotics, but there was no clinical or radiological response to treatment. Decision was made for a stereotactic biopsy. Intraoperative tissue cultures were negative for infection. However, histology reported B-cell lymphoma with morphological features changes typically seen in corticosteroid-treated lymphoma - an unexpected finding as no steroids were administered as part of her treatment. Owing to the unusual diagnosis, the case is presented in corroboration with current literature.

## Introduction

Primary central nervous system lymphoma (PCNSL) is a rare, extranodal non-Hodgkin lymphoma accounting for up to 6% of all primary brain tumors [1,2]. While it has been reported in immunocompetent hosts, its incidence is much higher in immunosuppressed patients and is frequently associated with HIV infection, post-transplantation, and other conditions related to chronic immunosuppression [3,4]. The authors report an unusual case of PCNSL masquerading as multifocal intracerebral abscesses in a patient on long-term treatment for myasthenia gravis (MG). In view of the unexpected diagnosis, the condition is discussed in collaboration with current literature and management strategies.

## Case presentation

An 89-year-old female presented with progressive functional decline of two weeks’ duration. Prior to this, she was fully independent in her activities of daily living and ambulated without walking aid. There was no family history of malignancy, recent travel, or contact history. During this period of decline, her family members noted global memory impairment, dysphasia, and increasing gait and swallowing difficulties. The patient had a background of non-thymomatous generalized seropositive MG and was given a course of prednisolone and azathioprine. Both medications were tapered off after a period of sustained remission. After a subsequent myasthenic crisis, she was started on mycophenolate mofetil (MMF) and pyridostigmine. She recovered well and was in remission since. At the time of this report, the patient had been on long-term MMF and pyridostigmine for more than 20 years (Table 1).

**Table 1:** List of patient’s long-term medications prior to her admission.

Medication	Dosage	Frequency
Alendronate	70 mg	Once weekly
Mycophenolate mofetil	500 mg	Twice daily
Pyridostigmine	60 mg	Thrice daily
Omeprazole	40 mg	Once daily
Atorvastatin	40 mg	Once daily
Aspirin	100 mg	Once daily
Isosorbide mononitrate controlled-release	30 mg	Once daily

Clinical examination did not demonstrate any obvious visceral or neurological abnormality. During physical examination, the patient was alert, non-toxic looking, and oriented to place and person, although she had a short attention span and slowed processing. There were no focal neurological deficits, meningism, or signs of raised intracranial pressure. Her laboratory results were largely unremarkable for any significant infective, metabolic, or systemic abnormalities (Table 2).

**Table 2:** List of laboratory investigations and their corresponding results. MCV, mean corpuscular volume; MCH, mean corpuscular hemoglobin

Laboratory investigations	Result	Normal range
Full blood count		
Total white cell count	4.5 x 10^9^/L	4.0 to 9.6 x 10^9^/L
Neutrophils	2.96 x 10^9^/L (65.6%)	1.90 to 6.60 x 10^9^/L
Lymphocytes	0.66 x 10^9^/L (14.7%)	1.10 to 3.10 x 10^9^/L
Monocytes	0.78 x 10^9^/L (17.2%)	0.20 to 0.70 x 10^9^/L
Eosinophils	0.08 x 10^9^/L (0.08%)	0.00 to 0.60 x 10^9^/L
Basophils	0.03 x 10^9^/L (0.03%)	0.00 to 0.10 x 10^9^/L
Hemoglobin	10.5 g/dL	11.8 to 14.6 g/dL
MCV	90 fL	83 to 98 fL
MCH	29 rg	28 to 34 rg
Platelets	179 x 10^9^/L	150 to 360 x 10^9^/L
Renal panel and electrolytes		
Sodium	134 mmol/L	135 to 145 mmol/L
Potassium	3.7 mmol/L	3.5 to 4.5 mmol/L
Serum creatinine	56 mmol/L	40 to 75 mmol/L
Urea	4.7 mmol/L	2.0 to 6.5 mmol/L
Corrected calcium	2.21 mmol/L	2.15 to 2.50 mmol/L
Magnesium	0.8 mmol/L	0.7 to 1.0 mmol/L
Phosphate	1.2 mmol/L	0.8 to 1.4 mmol/L
Liver function test		
Total bilirubin	8 µmol/L	5 to 30 µmol/L
Alanine transferase	26 U/L	5 to 40 U/L
Aspartate transferase	38 U/L	15 to 40 U/L
Alkaline phosphatase	88 mg/L	40 to 120 mg/L
Endocrine investigations		
Free thyroxine	13 rmol/L	8 to 16 rmol/L
Thyroid-stimulating hormone	1.88 mIU/L	0.45 to 4.50 mIU/L
Cortisol (taken at 08:00 hours)	315 mmol/L	240 to 618 mmol/L
Inflammatory markers		
C-reactive protein	0.6 mg/L	0.0 to 5.0 mg/L
Procalcitonin	0.06 µg/L	0.00 to 0.05 µg/L
Erythrocyte sedimentation rate	2 mm/hour	3 to 15 mm/hour

**Figure 1:**
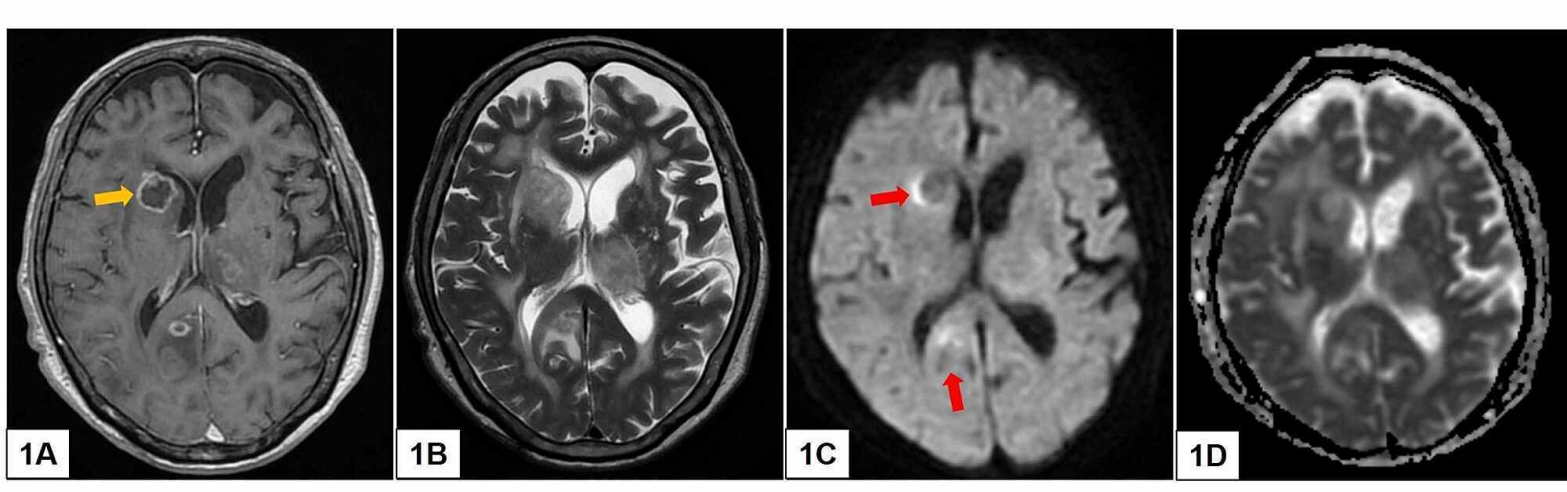
Representative MRI images in axial view in T1-weighted, post-contrast (A), T2-weighted (B), DWI (C), and its corresponding ADC map (D). Here, the imaging shows multiple, ring-enhancing intraparenchymal lesions of different sizes (A). Some of the lesions are associated with vasogenic edema (B). There is also evidence of restricted diffusion (red arrows) in (C) and (D). Of note, the ring-enhancing lesion adjacent to the right frontal ventricular horn (yellow arrow) is selected for the subsequent stereotactic biopsy. DWI, diffusion-weighted imaging; ADC, apparent diffusion coefficient

Magnetic resonance imaging (MRI) of her brain reported multiple, variably sized, ring-enhancing parenchymal lesions in the periventricular and lobar regions. There was associated perilesional vasogenic edema and local mass effect and gyral swelling. Some of the lesions displayed contents with restricted diffusion on diffusion-weighted imaging (DWI) (Figure 1). A follow-up computed tomography (CT) of her thorax, abdomen, and pelvis showed no other lesions of significance.

The imaging findings, in concert with the clinical presentation, were concerning for several etiologies given her history of long-term immunosuppressant therapy and radiological findings of multiple ring-enhancing lesions, the working diagnosis was that of an infective cause such as multiple cerebral abscesses versus cerebral toxoplasmosis. Another possibility based on her age, multifocal nature of lesion, and degree of vasogenic edema was that of a neoplastic process. Nonetheless, comprehensive CT imaging of her thorax, abdomen, and pelvis was performed as part of the workup. The results were negative for infective lesion or tumor growth. Extensive microbiological investigations including microbiological cultures and serological studies, including a transthoracic echocardiography, were unremarkable (Table 3). A lumbar puncture was not attempted in view of possible raised intracranial pressure.

**Table 3:** Microbiological investigations performed as part of infection workup. Ag, antigen; IgG, immunoglobulin G; HIV, human immunodeficiency virus; PCR, polymerase chain reaction; PAS, periodic acid-Schiff; GMS, Grocott methenamine silver

Investigations	Result
Relevant infection-related investigations	
Aerobic cultures (three sets)	No bacterial growth after 5 days
Anaerobic cultures (three sets)	No bacterial growth after 5 days
Acid-fast bacilli smear and cultures	Negative for mycobacterium species
Mycobacterium tuberculosis PCR	Negative
Cryptococcus neoformans Ag	Negative
Toxoplasma IgG	174 IU/mL
HIV serology	Negative
Transthoracic echocardiogram	Findings not suggestive of infective endocarditis (based on Duke’s diagnostic criteria)
Intraoperative tissue and fluid microbial investigations	
Aerobic cultures	No bacterial growth after 5 days
Anaerobic cultures	No bacterial growth after 5 days
Fungal cultures	No fungal growth
PAS stain	Negative
GMS stain	Negative
Tuberculosis PCR	Negative
Ziehl-Neelsen stain	Negative

**Figure 2:**
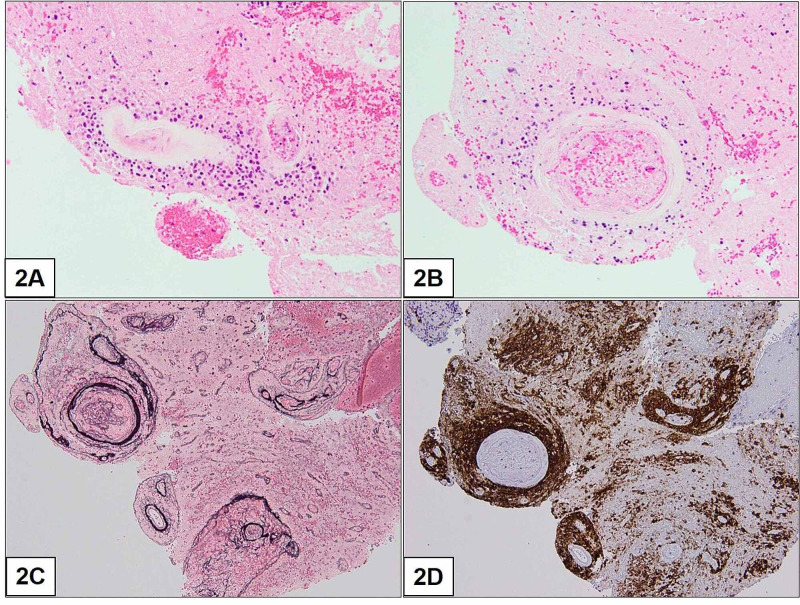
Histopathology images of lesion from stereotactic biopsy. (A and B) The necrotic areas in the biopsy show poorly preserved atypical cells adjacent to blood vessels (hematoxylin and eosin stained slides, x200). (C) The reticulin stain shows concentric networks of reticulin fibers around the blood vessels (reticulin immunohistochemistry stained slide x100). (D) CD20 is positive in the atypical cells. CD20 also highlights necrotic cells and shows granular staining in the necrotic areas (CD20 immunohistochemistry stained slide x100).

As multifocal cerebral abscesses were the top differential, the patient was started on meningitic doses of ceftriaxone and metronidazole. A repeat contrast-enhanced MRI of the brain was performed after a week of antimicrobial treatment, which revealed grossly stable size and appearance of the multiple ring-enhancing lesions with perilesional edema, and no new lesions were seen. In view of the lack of clinical and radiological improvement following a trial of antibiotics, stereotactic biopsy was performed to obtain definitive histopathological diagnosis. The right caudate lesion lateral to the frontal ventricular horn was selected for the procedure. A key consideration was the benefit of preventing its rupture (in view that its contents were possibly infective) into the CSF-filled ventricles. Intraoperative sampling from the lesion and its cystic component yielded firm necrotic tissue and small amounts of turbid fluid. These samples were sent for histological and microbiological examination. The intraoperative fluid and tissue aerobic and anaerobic cultures, fungal stains and cultures, TB (tuberculosis) stains, and TB PCR (polymerase chain reaction) tests were all negative. On histopathology, there was abundant necrotic tissue and inflamed granulation tissue. Within the necrotic tissue, atypical cells were seen around blood vessels. On immunohistochemistry, there were accompanying concentric networks of reticulin fibers around the blood vessels, and strong staining for CD20, a B-cell marker, in the atypical cells and necrotic cells (Figure 2). Putting it all together, these features were suspicious of B-cell lymphoma. Although our patient did not receive corticosteroids prior to biopsy, there were morphological changes similar to those observed in corticosteroid-treated PCNSL.

Putting it all together, these features were suspicious of B-cell lymphoma. Although our patient did not receive corticosteroids prior to biopsy, morphological features changes were observed in corticosteroid-treated PCNSL.

Owing to the patient’s advanced age and poor performance status, the option of curative chemotherapy was declined. Also, workup investigations such as positron emission tomography-CT (PET/CT) scans and bone marrow aspiration were not performed. Instead, decision was made for palliative whole-brain radiation therapy (WBRT). Antibiotics were discontinued, and the patient was maintained on low dose-dexamethasone during the duration of her WBRT (20 Gy in five fractions). She was discharged well with home hospice support.

## Discussion

PCNSL is an uncommon and malignant variant of extranodal non-Hodgkin lymphoma that involves the brain, leptomeninges, eyes, and spinal cord without evidence of systemic disease [4]. The location of the lymphoma in the central nervous system (CNS) typically determines the nature of clinical presentation [4]. Furthermore, patients often present in a subacute manner, with typical symptoms of altered mentation, cognitive decline, and signs secondary to raised intracranial pressure [5].

Although MRI is the radiological modality of choice, differentiating between PCNSL and cerebral abscess can be challenging. This is because ring-enhancing lesions on conventional MRI sequences have a list of differentials, ranging from infective processes to high-grade tumors [6]. Furthermore, cerebral abscess tends to show restricted diffusion on DWI sequences. For PCNSL in immunocompetent patients, their neoplasms are typically noted in periventricular areas and are often represented by contrast-enhancing lesions with vasogenic edema. They too can exhibit restricted diffusion on DWI [2,7]. Conversely, multilesional foci, ring enhancement, central necrosis and/or hemorrhage are reputed to be more common in immunosuppression-related PCNSL [1,8-10]. Advanced neuroimaging such as magnetic imaging spectroscopy (MRS) has been shown to be helpful in differentiating between PCNSL (whereby there are elevated lipid peaks and high Cho/Cr ratios) and cerebral toxoplasmosis in AIDS patients (where lipid/lactate peaks reflecting an anaerobic and necrotic inflammatory process are seen). Other metabolic imaging such as FDG-PET (fluorodeoxyglucose-positron emission tomography) and SPECT (single-photon emission CT) scans also have some utility in differentiating PCNSL (hypermetabolic) from infectious lesions (typically hypometabolic) [1,11]. In our patient, the presumptive diagnosis of multiple cerebral abscesses was not unreasonable, especially given the MRI findings of multifocal ring-enhancing lesions with central necrosis and perilesional vasogenic edema. Upon reflection of this unique case, the role of adjunct neuroimaging may have been useful for accurate diagnosis.

At this point in time, the association between iatrogenic immunosuppression and the development of PCNSL is unclear. We are already aware of how corticosteroids have a cytolytic effect on lymphoma cells [12,13]. This effect has been observed to be mediated by cytoplasmic receptors that, when bound to glucocorticoid, translocate to the nucleus and induce apoptosis [13]. Separately for MG, there are only a few case reports of patients diagnosed with PCNSL after long-term treatment in the literature [14,15]. In contrast, most MG patients do not develop PCNSL as a treatment-related complication. For instance, Herrlinger et al. reported two patients with MG who developed PCNSL after 6 and 12 years of azathioprine use, raising concerns of prolonged iatrogenic suppression of cell-mediated immunity leading to an increased risk of lymphoproliferative malignancy [14]. Similarly, Vernino et al published a case report of chronic MMF use in MG that was complicated by the development of PCNSL [15]. Here, the authors reported a patient with treatment regime for MG similar to ours. Furthermore, his patient also had similar lymphocytopenia and neuroimaging features of multiple ring-enhancing lesions, as in our patient. However, our patient’s histology was remarkably different. While the concentric perivascular arrangement and CD-20 stain positivity were preserved, the hypercellular and hyperproliferative features of PCNSL were largely attenuated with extensive necrosis seen. These features would be compatible with corticosteroid pre-treatment of PCNSL [16] but our patient did not receive any corticosteroids prior to biopsy. MMF is an inhibitor of inosine-5`-monophosphate dehydrogenase. It prevents synthesis of guanosine nucleotides preferentially in T and B lymphocytes and thus inhibits their proliferation [17]. Nonetheless, it is unknown if this particular effect of MMF can cause it to have a similar radiological presentation to a PCNSL pre-treated with corticosteroids.

Putting it all together, any subacute presentation of neurological deterioration, progressive lateralizing deficits, or signs and symptoms of raised intracranial pressure or meningism should prompt early neuroimaging, especially in high-risk patients. In immunocompromised patients, other important differential diagnoses such as CNS infections and other CNS malignancies should be considered. Neuroimaging findings may mimic other diagnoses such as cerebral abscesses. The use of advanced metabolic imaging can be helpful. Presently, histological diagnosis remains to be the gold standard and is paramount in guiding clinical treatment and management.

## Conclusions

In summary, the diagnosis of PCNSL requires a high index of suspicion, especially in patients on chronic immunosuppressants. The case in this report highlights the need for clinicians to be mindful of this rare entity as a differential diagnosis in a selected group of patients, as early intervention is paramount. We emphasize the need for international collaborations to work closely together for better, in-depth mechanistic understanding of this devastating disease.
